# Loss function influence on hyperparameter optimization for observational healthcare prediction models

**DOI:** 10.1093/jamia/ocag075

**Published:** 2026-05-14

**Authors:** Fleur Vereijken, Jenna M Reps, Peter Rijnbeek, Ross D Williams

**Affiliations:** Erasmus University Medical Center, Rotterdam, The Netherlands; Erasmus University Medical Center, Rotterdam, The Netherlands; Janssen Research and Development, Titusville, NJ, United States; Erasmus University Medical Center, Rotterdam, The Netherlands; Erasmus University Medical Center, Rotterdam, The Netherlands

**Keywords:** machine learning, hyperparameter tuning, Area Under the Curve (AUC), evaluation metrics, predictive modelling

## Abstract

**Objectives:**

Prediction models are increasingly used in healthcare for risk stratification and personalized care. Many models are developed using machine learning, which requires tuning hyperparameters to maximize performance based on a chosen loss function metric. In healthcare, the area under the receiver operating characteristic curve (AUROC) is commonly used for this purpose, but it may not always be the most appropriate choice for every clinical application. We empirically characterize whether the choice of loss function metric in hyperparameter optimization leads to systematic differences in model behavior across several clinical prediction tasks using real-world healthcare data.

**Methods:**

We utilized fifteen different loss function metrics to guide hyperparameter selection across three clinical prediction tasks and four machine learning algorithms. We then compared how loss function metric choice affected selected hyperparameters, overall performance, and individual predicted probabilities.

**Results:**

We observed that certain hyperparameters tended to have similar optimal values across different loss function metrics, although this pattern differed by algorithm. The best-performing models, evaluated using AUROC, were often not the models with hyperparameters optimized using AUROC. While models performed similarly at a population level, based on discrimination and calibration. The choice of the loss function metric had significant impact on the individual predicted risk for a patient.

**Discussion:**

The predictive multiplicity observed can have significant impact on the patient level, while not observed in the population level model evaluation.

**Conclusion:**

Predictive multiplicity can have a serious impact on patient treatment decisions but is not yet well understood.

## Introduction

Prediction models are increasingly used in healthcare research and practice, where they are used to analyze complex datasets to identify clinically relevant patterns and generate predictions. These models utilize patient records, including diagnostic information. The output is a probabilistic estimate of a future health outcome, often referred to as a prognostic prediction model. This model provides valuable insights for risk stratification and personalized patient care.^1^^,^^2^ These opportunities are promising. However, the implementation of complex data-driven models demands thorough evaluation. Their quality, relevance, and safety must be ensured before implementation.^3^

The performance of these algorithms is influenced by various factors, including data quality, quantity, feature selection, and, importantly, hyperparameter configuration.^4^ Hyperparameters, which influence a model’s complexity, behavior, and training speed, must be carefully tuned to achieve optimal performance.^5^ Typically, this involves first selecting the “optimal” hyperparameters for the model and then using these settings to learn the model’s parameters. Hyperparameters are configuration settings for machine learning models, such as the maximum tree depth or the number of samples to use when determining splitting rules in a decision tree. Unlike model parameters, which are learned and updated during the training process (eg, the specific splitting rules in a decision tree), hyperparameters are set before training begins and guide the learning process. As hyperparameters control aspects of the training procedure and model complexity, their values influence the parameter estimates learned during model training. Studies have consistently shown that tuning hyperparameters can significantly improve model performance over using default settings provided by common machine learning libraries.^6^^,^^7^

In general, a search strategy is implemented to identify the “optimal” hyperparameters for a model. This is accomplished by defining a set of possible hyperparameter combinations and evaluating how well each model with a different hyperparameter combination performs. Let *A*(*λ*) denote a learning algorithm that is parameterized by the hyperparameter vector *λ*. Additionally, there is a performance evaluation function ℳ, which assesses the algorithm’s effectiveness on a given learning task. The response function, ƒ(*λ*) = ℳ (*A*(*λ*)), combines these two components. The goal of hyperparameter optimization is to find the optimal values of *λ* that maximize or minimize ƒ(*λ*).^8^ Grid-search cross-validation is a common method for tuning hyperparameters.^9^ The user defines a set of possible values, forming a grid of all combinations. For each combination, cross-validation is performed: the training data were split into N folds, and the model is trained on N − 1 folds and evaluated on the held-out fold per fold. Performance is averaged across all folds to select the best hyperparameters. To assess this performance, a metric needs to be chosen for the evaluation. The area under the receiver operating characteristic curve (AUROC) is commonly used (ℳ in the functional relationship) for calculating this performance;^10–14^ however, it may not align with clinical priorities. For instance, AUROC measures how well the model can discriminate between classes but does not take class prevalence into account.^15^ Plus, when models are implemented as classifiers using a specified decision threshold, AUROC alone might not fully reflect clinical decision requirements.^16^ In such settings, threshold dependent metrics, such as precision, recall may better capture performance relevant to clinical use.

While hyperparameter optimization (HPO) has been widely studied in the machine learning and computer science literature, most prior work has focused on the efficiency and performance of different optimization strategies (eg, Bayesian optimization, random search, evolutionary methods) across various tasks and datasets.^17–20^ In these studies, tuning hyperparameters substantially enhanced model performance. However, far less attention has been given to the role of the *loss function* used during HPO. Specifically, how different performance metrics used as HPO objectives may shape model behavior. In this sense, HPO can also be viewed as a form of model selection, where different hyperparameter configurations correspond to different candidate models. In clinical contexts, the choice of loss function used during HPO is not neutral. It shapes which models are selected and how they behave in practice. Probability-based metrics (eg, Brier score, log loss) evaluate calibration, while threshold-based metrics (eg, F1 score, precision, recall) emphasize classification accuracy at specific decision boundaries. The implications of these choices are especially critical when the cost of misclassification is asymmetric, as is often the case in healthcare.

Because the optimization objective determines which model configurations are selected, the resulting parameters and hyperparameters depend on the chosen HPO objective. While this dependence is established in optimization theory,^21^^,^^22^ its practical implications for clinical prediction modeling pipelines have received limited empirical attention. We therefore hypothesize that the HPO objective will largely influence prediction model behavior in terms of classification errors, calibration, and discrimination. This study empirically characterizes and assesses the influence of metric selection in HPO for healthcare ML applications and highlights the trade-offs involved in aligning optimization with clinical utility.

## Methods

We investigate the impact of different loss functions versus a default loss function (AUROC) in hyperparameter grid search cross-validation using; multiple ML-algorithms, multiple performance measures (discrimination, calibration, and error measures), and multiple clinical prediction tasks.

We aim to determine:

1. How sensitive the optimal hyperparameters are to the choice of loss function
2. How sensitive the model performances are to the choice of loss function
3. How stable individual risk predictions are based on the choice of loss function.

### Loss function

We used fifteen different metrics (listed in Supplementary File 1, available as supplementary data at *Journal of the American Medical Informatics Association* online). During hyperparameter optimization, each metric was used as loss function metric to select the hyperparameter configuration that maximized or minimized the metric value in cross-validation. After selecting the optimal hyperparameters for each loss function metric, the resulting models were evaluated using these metrics as performance metrics to compare discrimination, calibration and classification errors across models.

### Database

The database used here is The Integrated Primary Care Information (IPCI) database.^23^ IPCI is a database containing longitudinal, routinely collected data from computer-based patient records of around 350 GP practices throughout the Netherlands. It is a dynamic database in which patients are included from their registration at the GP practice until death or leaving the practice. Every half year, a new version of the database is released. In total, the database currently (July 1, 2021) contains 2.5 million patients records with a median follow-up duration of 4.8 years. The number of active patients is 1.4 million, which comprises 8.1% of the Dutch population of 17 million. Data includes patient demographics, information about contacts with GPs, symptoms, diagnoses, laboratory and clinical measurements, prescriptions and information on use of secondary care. The primary goal of IPCI is to enable medical research. Since 2019 the data have been standardized to the Observational Medical Outcomes Partnership common data model (OMOP CDM),^24^ enabling collaborative research in a large international network of databases using standardized analytics. The IPCI database is under control of a Governance Board.

### Study prediction tasks

We selected three diverse clinical prediction tasks that differ in outcome prevalence, including low-prevalence settings where AUROC is known to be less informative,^15^ to assess the impact of hyperparameter optimization across clinically distinct datasets. Each prediction task consists of a target cohort (the patients we wish to make a prediction for), an outcome (what we are predicting), an index date (entails the cohort entry event, initial event, of time when people enter the cohort), and a time-at-risk (when we are predicting the outcome relative to the target cohort index).

### Prediction task 1, predicting dementia

The first prediction task’s target cohort included individuals aged 55–84 and the study period is between January 1, 2014, and December 31, 2014, ensuring that the 5-year follow-up period concluded by December 31, 2019. Hereby avoiding potential irregularities in the data caused by the COVID-19 pandemic. The earliest recorded visit to a healthcare provider is here the index date. Participants were required to have at least 365 days of continuous observation time before the index date (excluding the index date) for the assessment of candidate predictors.

The outcome is dementia onset within the 5-year period following the index date, focusing on individuals with no prior history of dementia.

### Prediction task 2, predicting lung cancer

The second predictions task’s target cohort included patients with an outpatient visit in 2017 with no prior cancer or screening for suspected cancer (first visit in 2017). Patients are included with ages between 45 and 65 years old. And the outcome was lung cancer within the 3-year period following the index date. A model for lung cancer using observational data is likely to be used to identify higher risk individuals that should be screened more regularly for lung cancer.

### Prediction task 3, predicting colorectal cancer

The third prediction task’s target cohort includes patients with anemia (date of first anemia record). With their outcome as colorectal cancer within the 1-year period following the index date.

### Algorithm choice

The different algorithms used in this research are Decision Tree (DT), Adaboost (Ada), Gradient Boosting Machine (GBM), and Light Gradient Boosting Machine (LGBM).

### Hyperparameter optimization

For hyperparameter optimization, we use cross-validation grid-search. For each algorithm we specified a grid of frequently used hyperparameters, listed in Supplementary Table 1, available as supplementary data at *Journal of the American Medical Informatics Association* online.

Within the grid-search we used fifteen different loss function metrics to calculate the “optimal” set of hyperparameters. Resulting in fifteen models per algorithm. The performance metrics used in the loss function can be divided into metrics that do not require a threshold to calculate their value, here referred to as probability-based performance metrics. And metrics that do require a threshold to calculate their value, hereafter referred to as threshold-based performance metrics. For threshold-based performance metrics we set the decision threshold to match the observed outcome rate in the dataset. This ensures that the proportion of positive predictions aligns with the outcome prevalence and provides a standardized reference for comparing models optimized under different objectives. Threshold selection is a design decision that should ideally reflect the clinical decision context and utility considerations.^25^^,^^26^ Here, the threshold was fixed to enable consistent comparison across optimization objectives.

An overview of all metrics used, can be seen in Supplementary File 1, available as supplementary data at *Journal of the American Medical Informatics Association* online.

### Model development

**Figure 1.**
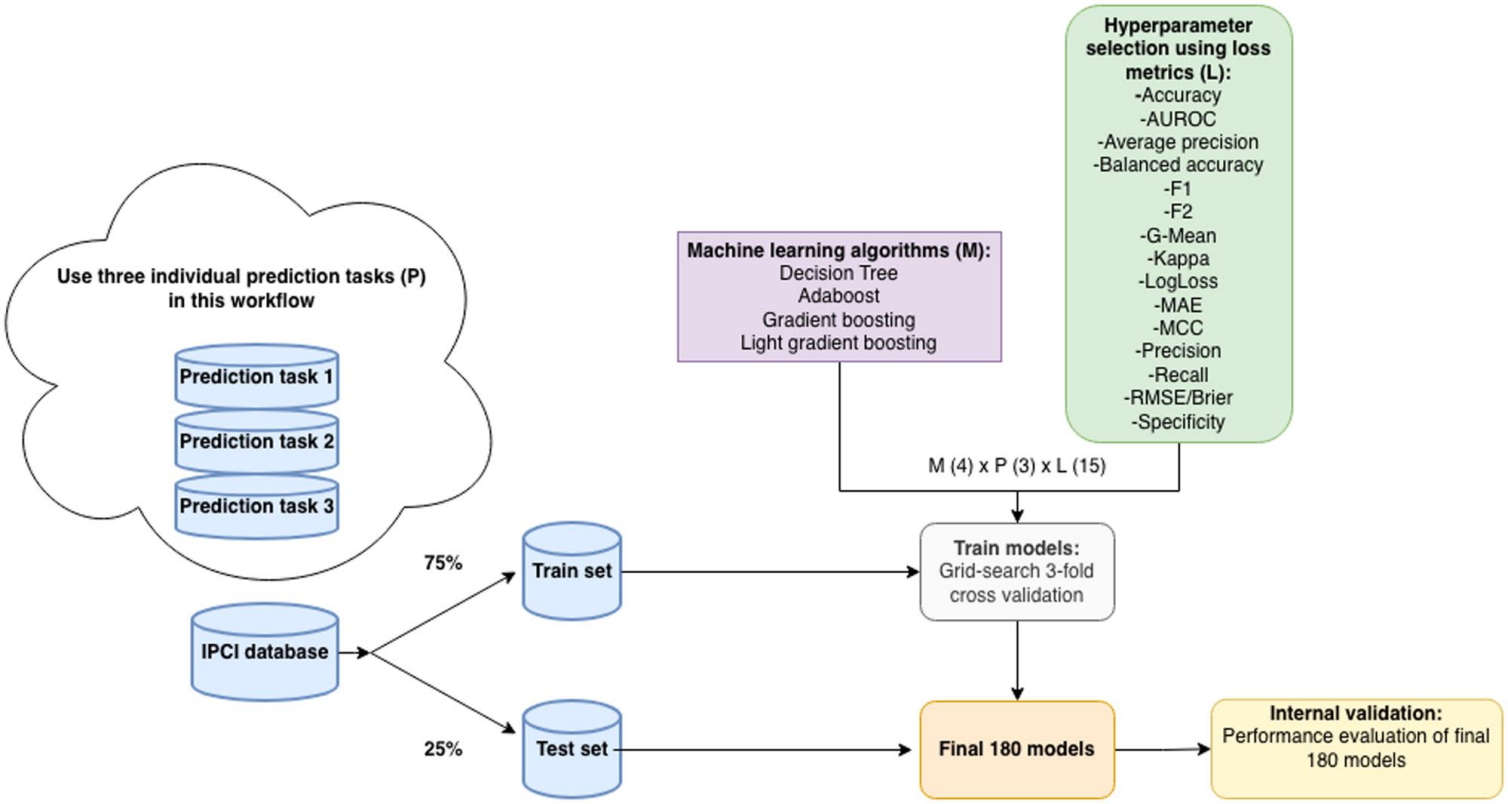
Overview of the workflow. Where we use three different prediction tasks, from the IPCI database. These will be split into training and test sets. All three prediction tasks will be trained using fifteen different loss function metrics using four different machine learning algorithms. All these models will be internally validated.

We developed our models using the OHDSI patient-level prediction framework.^27^ The data were partitioned into a training set consisting of 75% of these data and a test set consisting of the remaining 25%. Within the training data, threefold cross validation, which has been shown to be sufficient for large datasets,^28^ was used to select the optimal hyperparameters per performance metric. The final models were then trained on the full training set and evaluated on the independent test set (overview in Figure 1).

In our analysis, the model’s candidate predictors included all recorded conditions/drugs from the database’s condition and drug tables (one-hot encoding for all medical conditions/drugs that any patient had in the year prior to index), along with demographic variables such as age groups at index and sex. As the predictors were “had condition X recorded in prior 365 days” there were no missing values since a patient either has or does not have the condition recorded.

### Evaluation

#### Hyperparameter stability

We examined how the selected hyperparameters changed when different loss functions were used across algorithms. To assess whether different loss functions yield similar hyperparameters within certain algorithms, we performed clustering using Gower distance,^29^ a similarity measure suitable for mixed data types, including categorical and continuous values. We determined the optimal number of clusters using the silhouette score and visualized the results with multidimensional scaling (MDS).

#### Performance stability

To assess how sensitive performance is to the impact of using different hyperparameter optimization loss functions, we evaluate model performance across multiple dimensions. First model assessment will be performed using metrics that are most often used to evaluate prediction models; discrimination (AUROC) and calibration (Eavg). Eavg (0—inf) is the average absolute difference between observed and predicted probabilities.^30^

Next, the models will be evaluated on all performance metrics that are used as loss function metrics. For threshold dependent metrics, the threshold will be set at the average predicted risk, thus, varying per model.

#### Individual risk stability

To evaluate the sensitivity of individual risk predictions to the choice of hyperparameter optimization loss function, we will use a variant of level four stability; stability in a model’s predictions for individuals.^31^ Where we use the model with loss function metric AUROC as the reference model, because this is the most frequently used loss function metric, and all other models as comparator models. We also include average precision as a reference model, because of the class imbalance present in the datasets. We evaluate the individual predicted risks from each model by comparing them to the “default” model using the R^2^ metric. The R^2^ (coefficient of determination) measures how well one set of predictions matches another by quantifying the proportion of variance in the reference predictions explained by the model being evaluated. An R^2^ of 1 indicates perfect agreement; 0 indicates no agreement between the predicted probabilities. We tested whether disagreement in predicted probabilities led to differences in classification. We applied the same threshold as before, the mean predicted probability. We then compared the classification agreement between the AUROC and average precision as reference models and all other models.

#### Code availability

Code availability: https://github.com/mi-erasmusmc/EvaluationMetricStudy/tree/main

## Results

### Cohorts

For the dementia prediction task, applying inclusion and exclusion criteria yielded 184 985 patients, of whom 6256 (3%) developed dementia within 5 years. To reduce training time while retaining a meaningful number of cases, the dataset was randomly sampled to 30 000 patients. This subsampling was done as a prior study showed models achieve near optimal performance when there are 1000 outcomes or more.^32^

Table 1 shows the final cohort and test set sizes, along with the decision threshold based on outcome prevalence.

**Table 1.** Summary statistics overview of the different prediction tasks.

	Cohort size	Candidate features	Outcome (%)	Test size	Test size outcome	Threshold
***Prediction task 1, predicting dementia***	30 000	1208	1029 (3%)	7499	257	0.03
***Prediction task 2, predicting lung cancer***	173 373	1248	1065 (0.6%)	43 342	266	0.006
***Prediction task 3, predicting colorectal cancer***	87 570	1280	1280 (1.5%)	21 892	320	0.015

### Hyperparameter stability

We evaluated the impact of the loss function metric on hyperparameter choice in different algorithms and prediction tasks. For this, we used 4 different algorithms, 3 different prediction tasks, and 15 different loss function metrics. This all resulted in 180 models, from which the hyperparameter choices can be found in Supplementary Table 1, available as supplementary data at *Journal of the American Medical Informatics Association* online.

**Figure 2.**
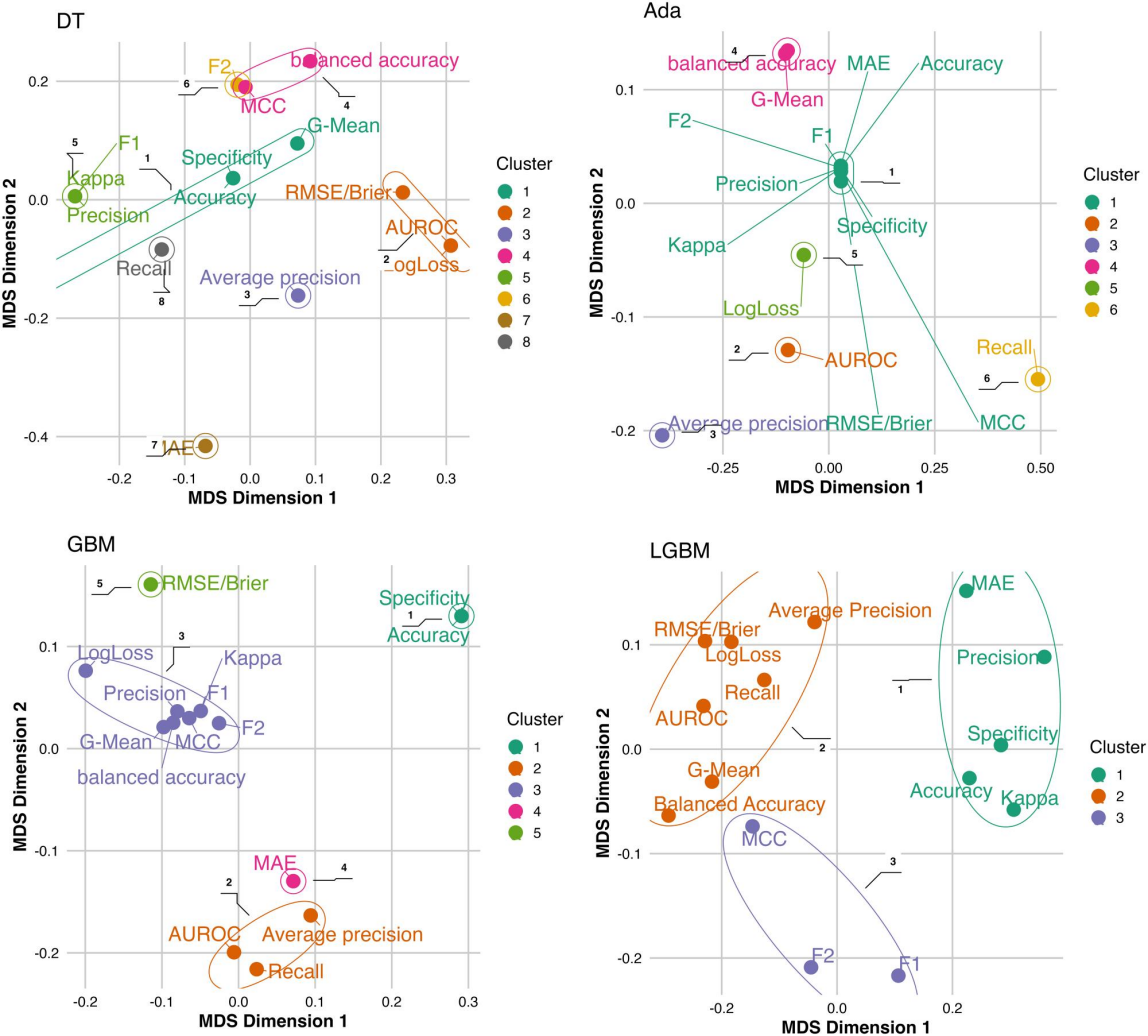
Multidimensional scaling for hyperparameter choice similarity. Per algorithm an overview of how similar the hyperparameter choices are per loss function metric used. There is no pattern observed between clustering of loss function metrics across the different algorithms.

Figure 2 shows for each algorithm how similar the loss function metrics are in terms of choosing the hyperparameters, aggregated over the three prediction tasks. Clusters indicate that loss function metrics often result in the same or similar hyperparameter choices. Across the different algorithms it is evident that different loss function metrics cluster together. In the Ada algorithm, almost all loss function metrics cluster together, except for loss functions average precision and recall. Whereas for the DT algorithm, loss function metrics result in more distinct hyperparameter choices. All hyperparameter choices are illustrated in Supplementary Figure 1, available as supplementary data at *Journal of the American Medical Informatics Association* online (prediction task 1), Supplementary Figure 2, available as supplementary data at *Journal of the American Medical Informatics Association* online (prediction task 2), and Supplementary Figure 3, available as supplementary data at *Journal of the American Medical Informatics Association* online (prediction task 3).

### Performance stability

**Figure 3.**
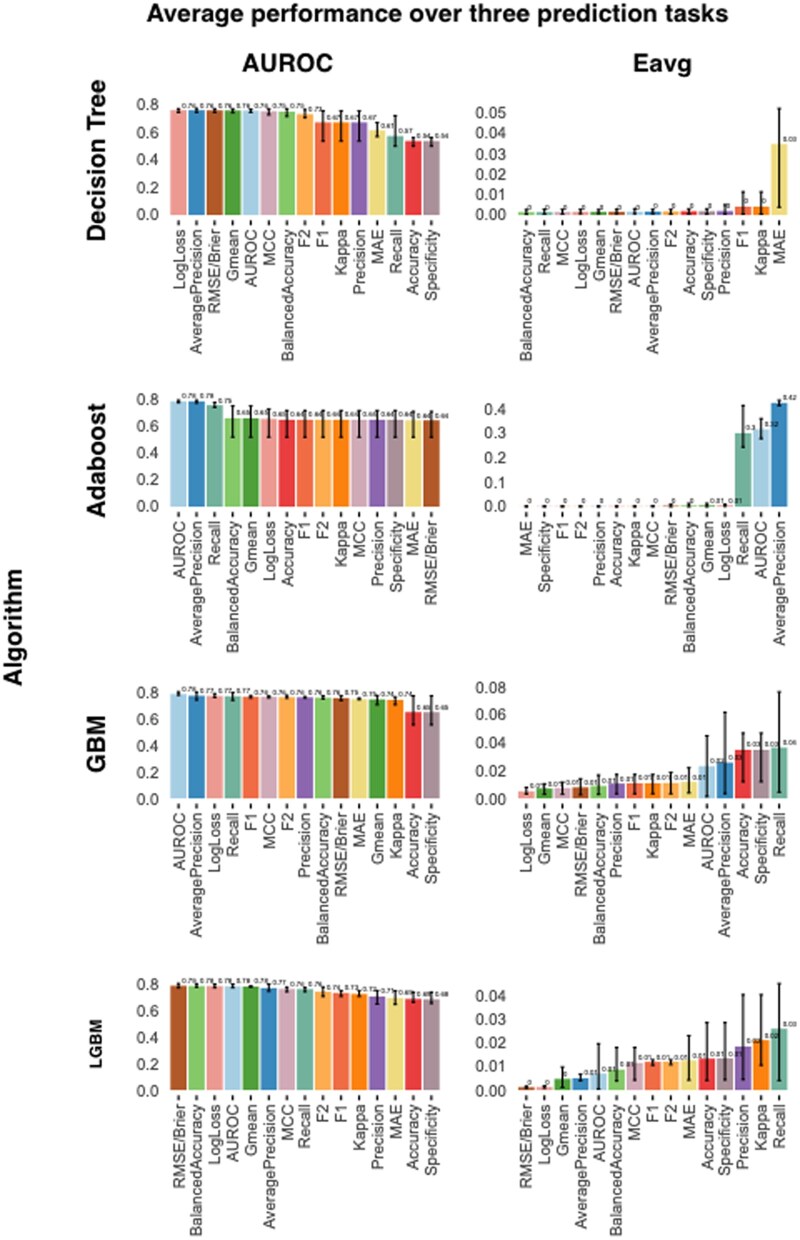
Barplot in decreasing performance order measured in AUROC and Eavg. Shown are the average scores across prediction tasks for the best models per algorithm, with intervals indicating the lowest and highest values achieved. The color coding is based on the loss function used. It can be observed that the optimal loss function for discrimination and calibration varies across algorithms.

Evaluation of prediction models often focuses on discrimination (AUROC) and calibration. Ideally, models should perform well on both. Figure 3 shows a bar plot in decreasing the best average AUROC (discrimination) and Eavg (calibration) scores across the three prediction tasks.

For DT, the LogLoss, Average Precision, RMSE/Brier, G-Mean and AUROC trained models scored similar on the discrimination (0.76), with only variation in decimal points. These five models show also similar performance measured in Eavg.

For Ada, the three best performing models (AUROC, Average Precision and Recall) on discrimination, showed to be the least calibrated models. The rest of the trained models showed at least a 10% decrease in discrimination performance but are well calibrated.

For GBM, the LogLoss-trained model performed well on both measures. For LGBM, the RMSE/Brier model achieved the best overall discrimination and calibration. The LogLoss and AUROC models also ranked among the top performers in both categories.

Supplementary Figure 4, available as supplementary data at *Journal of the American Medical Informatics Association* online provides an overview of all models in decreasing order of model performance, for all performance metrics used as loss functions.

### Individual prediction stability

**Figure 4.**
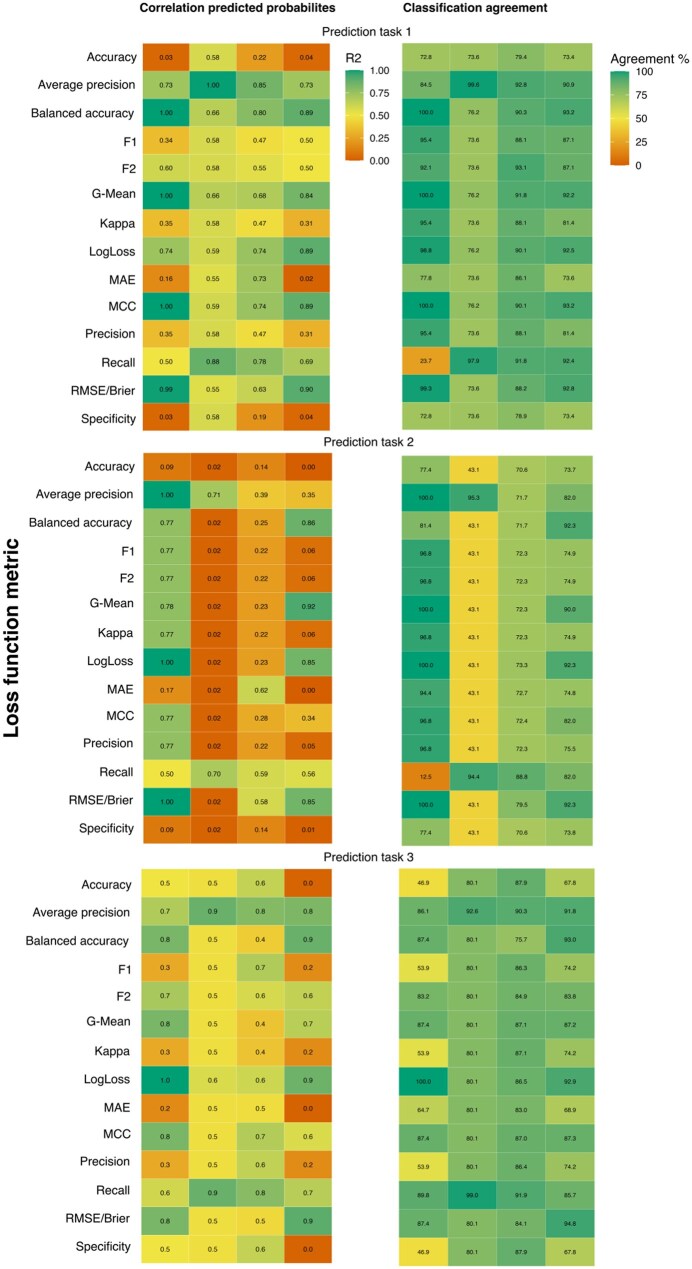
Heatmaps of agreement between models in predicted probabilities. The correlation between the predicted probabilities is expressed in R^2^. The classification agreement is expressed in %, indicating the percentage of patients where the reference model and the comparator model agree. The AUROC-optimized model was used as the reference, with all other models shown as comparators. The x-axis indicates the different algorithms, and the y-axis shows the comparator models. Even when predicted probabilities are weakly correlated across models, classification agreement can remain high.

Figure 4 shows the correlations in individual predicted probabilities (*R*^2^) between the AUROC model and all other models. We also show the correlation in individual prediction between average precision and other models (Supplementary Figure 5, available as supplementary data at *Journal of the American Medical Informatics Association* online). These figures also show the impact of the individual predicted probabilities if the model were to be implemented, thus the impact on the classification, expressed in classification agreement. It can be observed that many models differed strongly in their individual predictions compared to the AUROC and average precision models, yet if the models were to be implemented, >70% agreement in classification between the models is observed. The Ada models in prediction task 2 and some DT models across different prediction tasks did vary more on individual level.

In prediction task 1, models often shared similar AUROC but showed substantial differences in individual risk predictions, as indicated by R^2^. For example, in the GBM models for predicting colorectal cancer, the assigned risk, for one patient, could vary from 1.5% to 51%. Thus, models with comparable discrimination could still assign very different risks to patients. Even models with little correlation in predicted probabilities, such as DT models optimized for Accuracy and Specificity, often maintained >70% classification agreement. In prediction task 2, a DT model optimized for G-Mean reached an *R*^2^ of 0.78, yet classified all patients identically.

## Discussion

This study empirically characterized the stability of prediction models developed using observational healthcare data when hyperparameters are optimized using different loss functions. We demonstrated that using AUROC as a loss function for hyperparameter optimization may not always be the most suitable choice.

Regarding the stability in hyperparameter selection, the greatest variability was observed in DT, GBM, and LGBM. This indicates that these algorithms are more sensitive to the choice of loss function metric.

If both discrimination and calibration are important for the prediction model, the LogLoss loss function metric produced stable performance for these aspects in the Ada and GBM models. For the LGBM models, RMSE and Brier loss function metrics yielded the most optimal balance between discrimination and calibration. Interestingly, optimizing hyperparameters with a specific loss function metric did not necessarily produce the best performance on that same performance metric. For example, using AUROC as the loss function metric did not consistently result in the model with the highest discrimination. Separately, Van Calster et al.^33^ evaluated several performance metrics in terms of their properness and focus, defining properness as rewarding accurate probability estimates and focus as emphasizing performance in clinically meaningful regions of the score distribution. In their assessment, AUROC, the Brier score/RMSE, and LogLoss were the only metrics, that we used here, that satisfied both criteria, average precision was proper but not focused, and all remaining metrics were considered not proper.

Then, while several models seemed to perform similarly based on overall performance metrics (AUROC and calibration), these models varied on individual predictions; assigning different risks to the same individual. Meaning, model A and B have similar average performance scores, but model A assigns the risk for a single individual to be 1% and model B assigns a risk for the same individual to be 55%. This varying risk would dramatically impact treatment choice based on a decision boundary. This would mean that based on the choice of loss function metric alone, an individual would be treated differently. And even when applying a decision threshold and looking at how these individuals are classified, the classification agreement mostly was >70%. And even though this sounds like quite sufficient overlap between models, it still indicates, for prediction task 2, around 13 002 out of 43 342 individuals are differently classified, and therefore differently treated.

Therefore, it would be interesting to dive further into which patients are differently classified across certain models. In this context, McDermott et al.^34^ recently published a paper examining the behavior of AUROC and AUPRC under class imbalance. Their findings indicate that AUROC, when used as a loss function metric, treats all types of errors equally, whereas AUPRC places greater emphasis on high-score regions. In other words, if the costs of different errors are unknown and the goal is to train a model that weighs all mistakes the same, AUROC may be the more suitable choice. Conversely, if the costs of errors are known and accuracy in high-score regions is more important, AUPRC or average precision may be more appropriate as loss function metrics. Expanding this type of analysis to additional metrics could offer further insight into how different loss functions shape model preferences. For clinical applications, priorities may differ depending on the consequences of false positives and false negatives. When the goal is to identify as many at-risk individuals as possible, recall becomes a key performance criterion. In such cases, it is important to assess which loss function leads to models with stable discrimination, calibration, and recall. Although optimizing recall often produced models with the highest recall scores, this typically came at the expense of AUROC, calibration, or precision. This highlights a potential pitfall: recall favors identifying all at-risk patients, even if many are incorrectly flagged. Therefore, we advise against using extreme loss functions, such as recall, precision, accuracy, or sensitivity on their own. When these metrics are prioritized, we recommend applying constraints, such as optimizing recall while maintaining a minimum level of precision or adopting a multi-criteria optimization approach that explicitly balances competing performance objectives.^35–37^

The concept of similar performance metrics but with differing predicted probabilities aligns with the concept of predictive multiplicity.^38^ These findings show that overall performance stability can mask substantial variation in individual predictions. This affects clinical decision-making, where absolute risk values are important. Even if classification agreement appears high, many patients are classified differently when models use different loss function metrics. Behzad et al.^39^ proposed a new algorithm to address predictive multiplicity. Their approach leverages disagreements between models to falsify and improve at least one of them. Variation in predictions across models can also be viewed as uncertainty quantification. In statistical learning, parameter estimates and model outputs can be considered random variables influenced by data variability, model specification, and algorithmic choices. Methods from the uncertainty quantification literature, including conformal prediction and related approaches, aim to characterize and communicate such uncertainty in model predictions. Situating model variability within this broader framework may help better understand when differences between models reflect meaningful uncertainty versus instability in the modeling pipeline.^40–42^ Future work could further examine how uncertainty arising from hyperparameter selection affects prediction stability and downstream clinical decisions. Additionally, aligning optimization objectives with the clinical consequences of prediction errors may improve the interpretability and trustworthiness of machine learning models used in healthcare applications.^43^^,^^44^

## Limitations

In our experiments, the decision threshold for threshold-dependent metrics was fixed to the outcome prevalence. However, this threshold can itself be considered a hyperparameter. Future work could explore dynamic threshold selection strategies or incorporate threshold tuning directly into the optimization process. We here looked at using one loss function metric at a time for HPO optimization, while it would also be possible to use multi-criteria optimization.

All analyses were conducted using data from a single database. While this allowed for controlled comparisons, applying the same methodology to diverse datasets would help generalize the findings across different populations and clinical settings.

In addition, although we started to see trends, we only investigated the impact of hyperparameter loss function across three prediction tasks. In future work it would be interesting to apply our comparison to more prediction tasks to see whether the trends hold.

Finally, we used a grid search for hyperparameter tuning. While this is straightforward and interpretable, it may not capture optimal configurations as efficiently as more advanced methods like Bayesian optimization or random search. Future studies could evaluate whether the observed stability patterns hold under alternative optimization strategies.

## Conclusions

Overall, different loss function metrics strongly influence which hyperparameters are selected during model development. In the DT and Ada models it seemed to have the greatest impact based on the average performance metrics. So here it would be interesting to explore whether the loss function metric aligns with the specific clinical objective of the prediction task. While, for GBM and LGBM the effect of different loss function metric does not always appear to affect overall performance, based on average performance metrics. It does impact individual predicted probabilities and how patients are classified. Although the problem with individual predictions differing is hard to solve and needs further research to address. It only became evident that in every algorithm across every prediction task, the choice of loss function metric can meaningfully affect the interpretability of model predictions.

## Supplementary Material

ocag075_Supplementary_Data

## Data Availability

Fleur Vereijken is responsible for the data. The data that support the findings of this study are not publicly available due to privacy concerns regarding patient-level information.
