# Supplementary material for: Loss function influence on hyperparameter optimization for observational healthcare prediction models
Source: J Am Med Inform Assoc. 2026 May 14;33(8):1474–84. doi: 10.1093/jamia/ocag075 (PMC13386010; doi:10.1093/jamia/ocag075)
Supplement: ocag075_Supplementary_Data [file ocag075_supplementary_data.zip › Supplementary.docx]

**SUPPLEMENTARY**

***Supplementary file 1*** *Overview of all performance metrics used as loss functions. Containing their mathematical notation and explanation.*

**Probability-Based performance metrics**

Probability-based metrics assess the quality of predicted probabilities rather than binary classifications derived from applying a threshold. These metrics evaluate how well the predicted probabilities reflect the true likelihood of the target outcome. The probability-based performance metrics employed in this study include AUROC, Average Precision, LogLoss, Brier score, RMSE, and MAE. The mathematical notation of these metrics is notated below.

- **Area Under the Receiver Operating Characteristic Curve (AUROC):**

AUROC measures the probability that, for two randomly selected patients, the patient who experiences the outcome is assigned a higher predicted risk than the one who does not. Mathematically, it is calculated as:


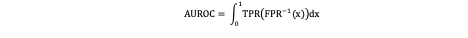


Where TPR (true positive rate) and FPR (false positive rate) are functions of the classification threshold.

- **Average Precision:**

Average precision quantifies the performance of a model that produces ranked prediction by evaluating how well it ranks the relevant items higher than irrelevant ones:


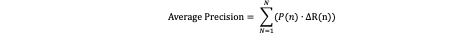


Where N would be the relevant instances, *P(n)* would be the precision at the *n*-th threshold and ΔR(n) the change in recall at the *n*-th threshold.

- **Logarithmic Loss (LogLoss):**
   LogLoss quantifies the accuracy of probabilistic predictions by penalizing predictions far from the true labels. It is defined as:


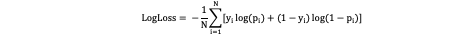


Where y_i_ is the true label (0 or 1), and p_i_ is the predicted probability for the positive class.

- **Brier Score:**
  The Brier Score evaluates the mean squared error between predicted probabilities and actual outcomes. It is calculated as:


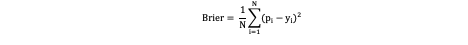


- **Root Mean Squared Error (RMSE):**
   RMSE measures the square root of the mean squared error of predictions:


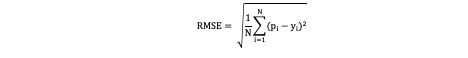


- **Mean Absolute Error (MAE):**
   MAE calculates the average absolute difference between predicted probabilities and true outcomes:


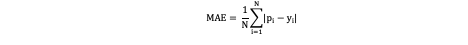


**Threshold-Based performance metrics**

Threshold-based performance metric need a decision threshold to classify predicted probabilities into binary outcomes. The threshold-based performance metric utilized in this study include accuracy, precision, recall, F1-score, specificity, MCC, balanced accuracy, G-Mean, kappa score and F2 score. Mathematical notation of these metrics is listed below.

- **Accuracy:**
  Accuracy measures the proportion of correct classifications out of all predictions:


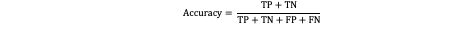


- **Precision:**

Precision (or Positive Predictive Value) evaluates the proportion of correctly predicted positive cases among all predicted positives:


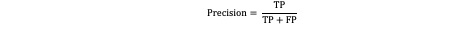


- **Recall (sensitivity):**

Recall (or True Positive Rate) measures the proportion of actual positives correctly identified:


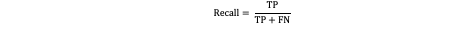


- **F1 Score:**
  The F1 score is the harmonic mean of precision and recall, balancing their trade-off:


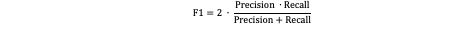


- **Specificity:**
  Specificity (True Negative Rate) measures the proportion of actual negatives correctly classified:


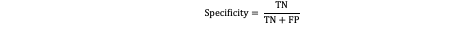


- **Matthews Correlation Coefficient (MCC):**
  MCC is a balanced measure that accounts for all confusion matrix categories and ranges from -1 to 1:


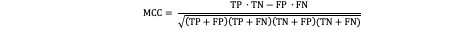


- **Balanced Accuracy:**
  Balanced accuracy averages sensitivity and specificity to account for class imbalance:


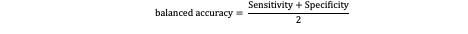


- **Geometric Mean (G-Mean):**
  G-Mean emphasizes the balance between sensitivity and specificity:


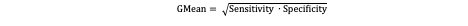


- **Cohen’s Kappa (Kappa):**
  Kappa measures agreement between predictions and actual outcomes while accounting for random chance:


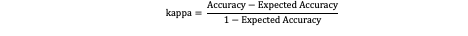


- **F2 score:**
  The F2 score prioritizes recall over precision by weighting recall twice as much as precision:


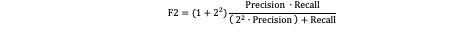


***Supplementary Table 1*** *Overview of the hyperparameter grid per algorithm.*

| ***Hyperparameter*** | **Decision Tree** | **Adaboost** | **GBM** | **LGBM** |
| --- | --- | --- | --- | --- |
| *Criterion* | (gini, entropy) |  |  |  |
| *Splitter* | (best, random) |  |  |  |
| *maxDepth* | (4, 10, 20, NULL) |  | (4, 6, 8, 10) | (5, 10, 15, -1) |
| *minSampleSplit* | (2, 5, 10) |  |  |  |
| *minSamplesLeaf* | (10, 50) |  |  |  |
| *maxFeatures* | (log2, sqrt, 100, NULL) |  |  |  |
| *minImpurityDecrease* | (10^-7, 10^-4) |  |  |  |
| *nEstimators* |  | (5, 10, 20, 50, 75, 100, 200, 300) |  |  |
| *learningRate* |  | (1, 0.5, 0.1, 0.01, 0.001) | (0.01, 0.05, 0.1, 0.3) | (0.01, 0.05, 0.1, 0.3) |
| *ntrees* |  |  | (100, 300, 500) |  |
| *minChildWeight* |  |  | (1, 3, 5) |  |
| *scalePosWeight* |  |  | (1, 10) | (1, 10) |
| *lamda* |  |  | (0, 0.1, 1, 5, 10) |  |
| *alpha* |  |  | (0, 0.1, 0.5, 1, 5) |  |
| *numIterations* |  |  |  | (100, 300, 500) |
| *numLeaves* |  |  |  | (31, 63, 127) |
| *minDataInLeaf* |  |  |  | (20, 50, 100) |
| *lambda1* |  |  |  | (0, 0.5, 1) |
| *lambda2* |  |  |  | (0, 0.5, 1) |

***Supplementary figure 1*** *Overview of chosen hyperparameters by loss functions per algorithm for prediction task 1.*

***Supplementary figure 2*** *Overview of chosen hyperparameters by loss functions per algorithm for prediction task 2.*

***Supplementary figure 3*** *Overview of chosen hyperparameters by loss functions per algorithm for prediction task 3.*

***Supplementary figure 4*** *Barplot in decreasing performance order measured in all performance metrics that also are used as loss functions. Shown are the average scores across prediction tasks for the best models per algorithm, with intervals indicating the lowest and highest values achieved. The color coding is based on the loss function used.*

***Supplementary figure 5*** *Heatmaps of agreement between models in predicted probabilities. The correlation between the predicted probabilities is expressed in R^2^. The classification agreement is expressed in %, indicating the percentage of patients where the reference model and the comparator model agree. The average precision-optimized model was used as the reference, with all other models shown as comparators. The x-axis indicates the different algorithms, and the y-axis shows the comparator models. Even when predicted probabilities are weakly correlated across models, classification agreement can remain high.*
