# Supplementary figures and images for: Loss function influence on hyperparameter optimization for observational healthcare prediction models
Source: J Am Med Inform Assoc. 2026 May 14;33(8):1474–84. doi: 10.1093/jamia/ocag075 (PMC13386010; doi:10.1093/jamia/ocag075)

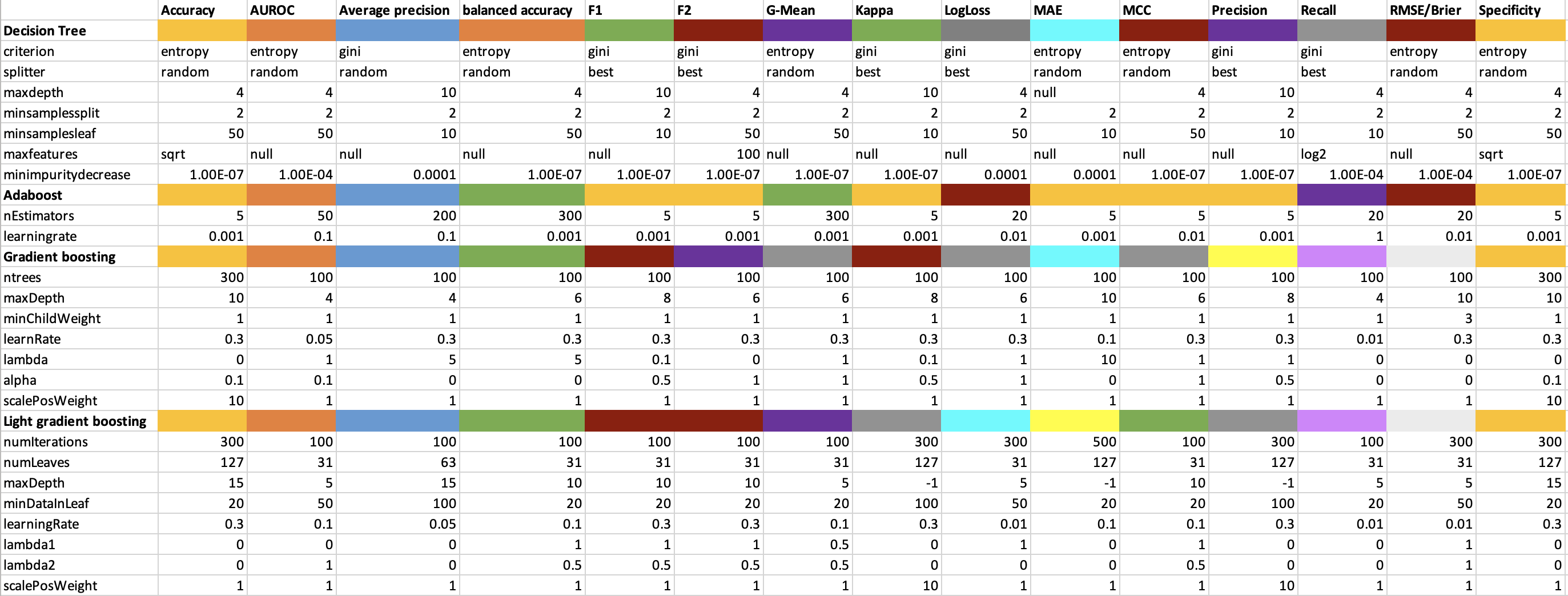

Supplement: ocag075_Supplementary_Data [file ocag075_supplementary_data.zip › SupplementaryFigure1.png]

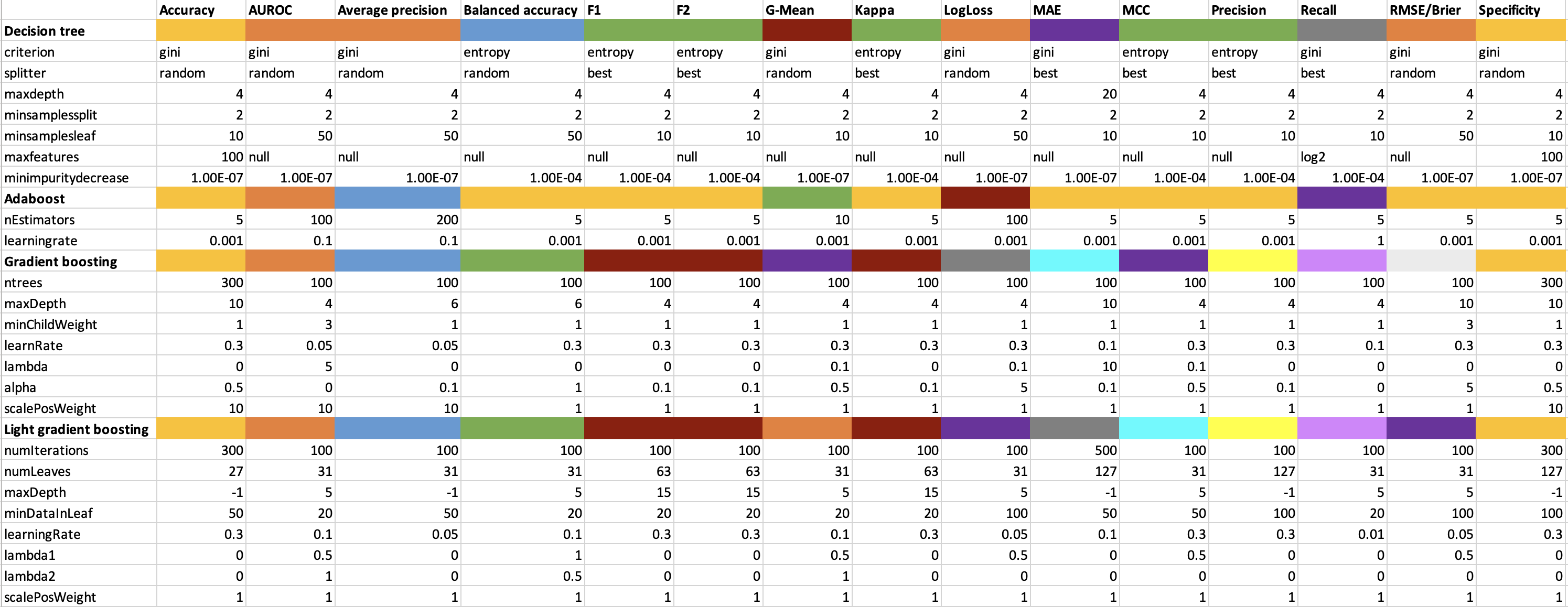

Supplement: ocag075_Supplementary_Data [file ocag075_supplementary_data.zip › SupplementaryFigure2.png]

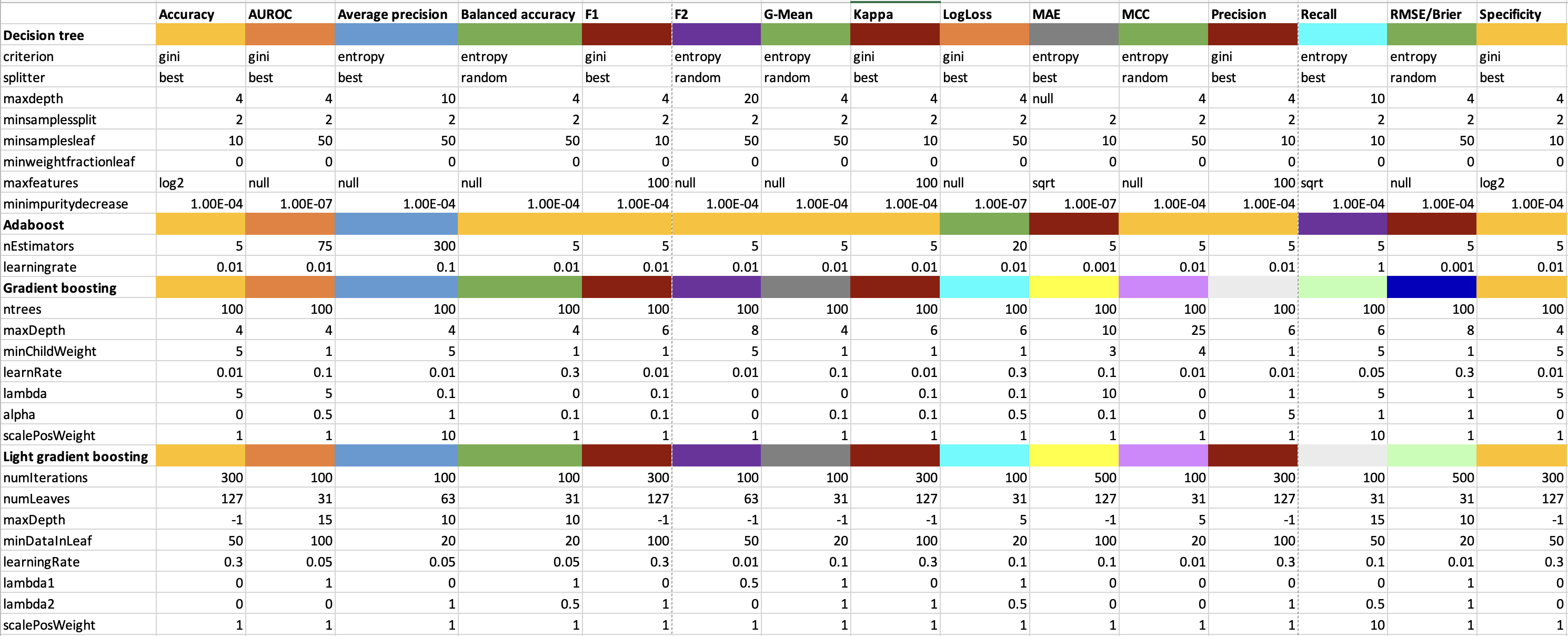

Supplement: ocag075_Supplementary_Data [file ocag075_supplementary_data.zip › SupplementaryFigure3.png]

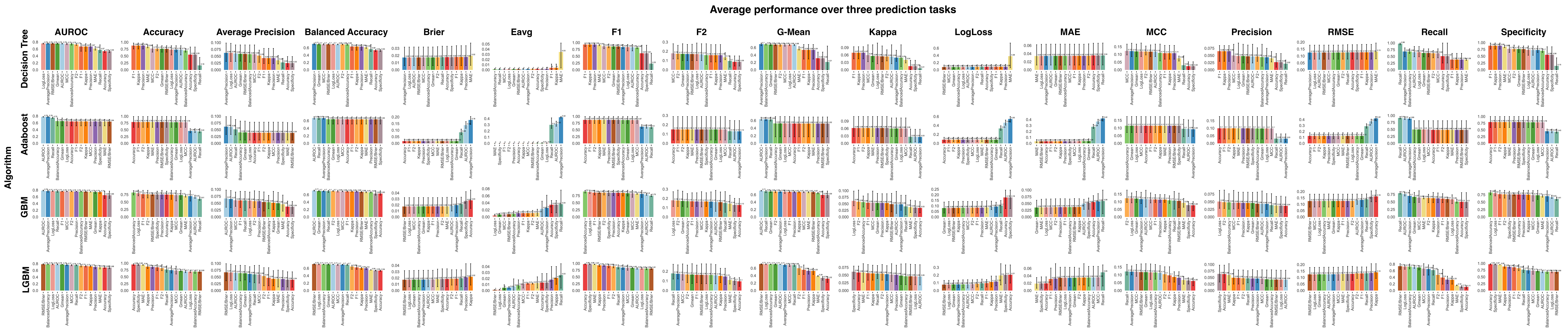

Supplement: ocag075_Supplementary_Data [file ocag075_supplementary_data.zip › SupplementaryFigure4.png]

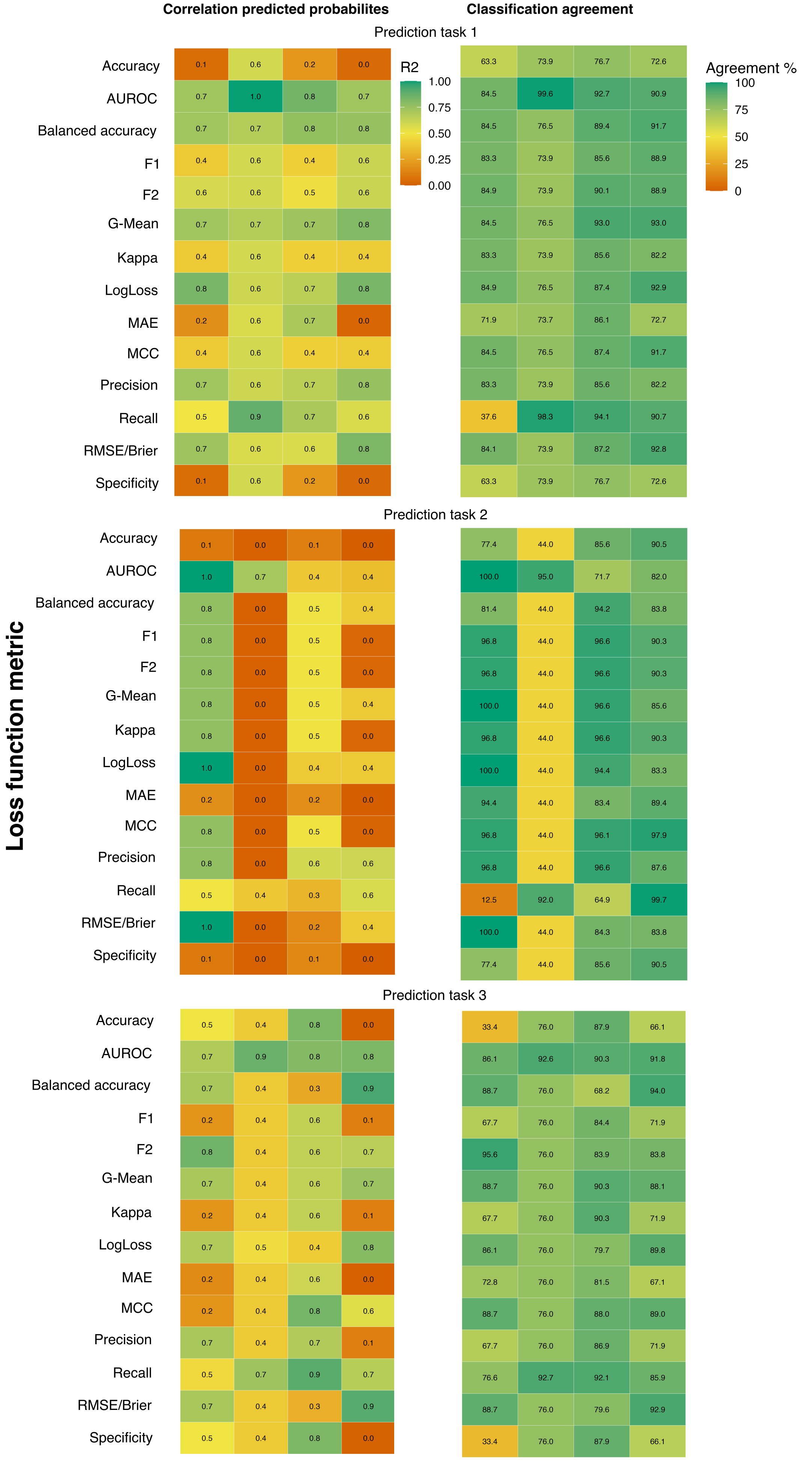

Supplement: ocag075_Supplementary_Data [file ocag075_supplementary_data.zip › SupplementaryFigure5.png]
